# A systematic databasing of diatoms from different geographical localities and sites of Haryana for advancing validation of forensic diatomology

**DOI:** 10.1016/j.dib.2016.11.072

**Published:** 2016-11-24

**Authors:** Ekta Saini, V.P. Khanagwal, Rajvinder Singh

**Affiliations:** a Department of Genetics, M.D. University, Rohtak 124001, Haryana, India; b Department of Forensic Medicine, Pt. B.D.S. University of Health Sciences, Rohtak 124001, Haryana, India

**Keywords:** Drowning, Diatoms, Water body, Microscopy

## Abstract

Verdict on tracing exact place of drowning is a part of medico-legal investigation. This question often stands when circumstances remain unclear about true drowning place. Usually, when a dead body rises from the bottom of drowning site, it will appear near to the point where it had actually disappeared but rapid current may carry a body to real distance from the exact place of death before any major obstruction. Forensic methodology has suggested qualitative as well as quantitative comparison of diatoms recovered in dead body and reference water samples to corroborate drowning as cause of death and locating precise place of drowning. Collection of wrong reference water samples from drowning site can also hamper the investigation process. Since, the distributions of different genera in certain extents relate particular water where the death due to drowning might have taken place; therefore, the present attempt was made to understand diatom distribution in five water bodies of Haryana with reference to diatom growth factors. This research data represents diatomological profiles of selected sites for possible application of forensic diatomology. Both, the light and scanning electron microscopy identified diatoms. It is envisioned that this data report is informative enough for the experts to plan future strategy for investigating mysteries associating place of drowning.

**Specifications Table**

**Table:** 

Subject area	Forensic Science
More specific subject area	Forensic Diatomology
Type of data	Figure and Tables
How data was acquired	Identification of diatoms was made with light and scanning electron microcopy (Leo 435 VP)
Data format	Analyzed
Experimental factors	Water samples collected in different seasons were treated with acid digestion method, and later centrifuged to extract diatoms
Experimental features	The distribution patters of diatoms in selected water bodies were evaluated with context to seasonal and geographical changes
Data source location	Haryana (29.0588°N, 76.0856°E) a northern state of India
Data accessibility	Data is available with this article

**Value of the data**

• Little is known about the diatomological mapping of fresh water bodies of Haryana.
• Diatomological information produced here provides a baseline data which can aid future efforts in investigation of the diatom diversity present in Haryana.
• Data provide details of the strategy for the forensic and medicolegal experts to trace the informative pathway dealing with questioned drowning place.
• These data are also useful for researchers working in the field of archaeology, botany and environment sciences for water quality assessment and environmental change.

## 1 Data

**Fig. S1:**
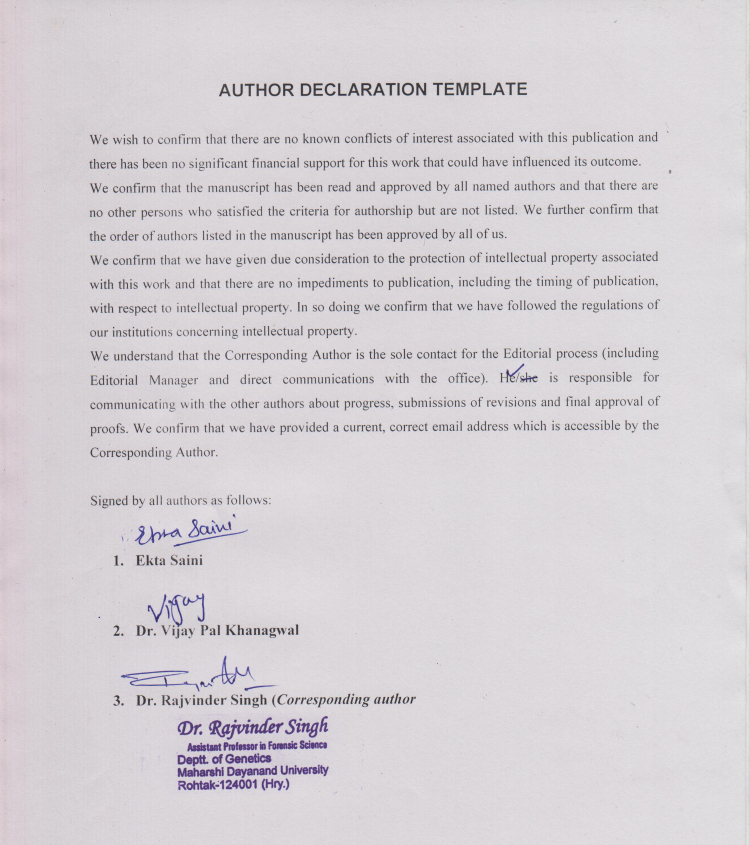


Data provided in this article revealed diversity of diatom depending upon properties of habitat water and prevailing climatic conditions of water bodies from varied localities of Haryana (Supplementary Fig. 1).

## 2 Experimental design, materials and methods

Water samples were collected from Morni Hills Tikkar Taal Lake, Panchkula; Tilyar Lake, Rohtak; Jawaharlal Nehru Canal, Rohtak; Kharawad Village Well, Rohtak; and Suraj Kund, Faridabad. The first sampling was conducted during winter season in the month of December 2013. The schedule was continued following spring (March), summer (June) and autumn (September) seasons in 2014. Changes in the physical characteristics of these water bodies have been depicted in Table 1. Temperature and pH of water were also recorded at the time of sampling (Table 2, Table 3).

**Fig. 1:**
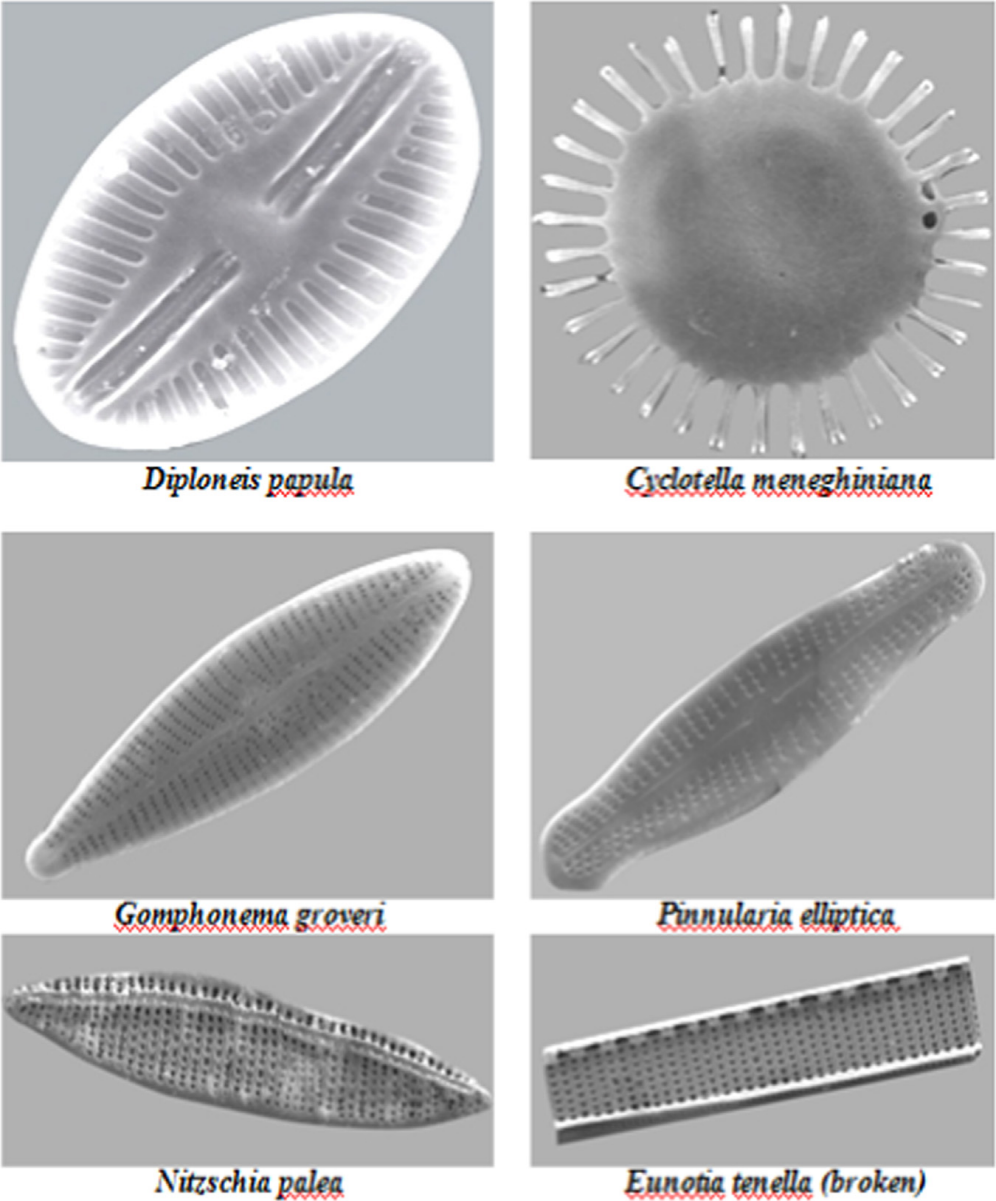
Photomicrographs of diatoms through Scanning Electron Microscope.

Earlier mentioned protocols [1], [2] were followed right from the extraction up to qualitative and quantitative analysis of diatoms. Diatoms were identified on the basis descriptions available in the literature [3]. Distribution patterns of diatoms have been displayed in Table 4. Photomicrographs of some diatoms can also been viewed in Fig. 2 (Supplementary) and Fig. 1.

## Figures and Tables

**Table 1:** Physical characteristics of studied water bodies.

**Name of water body**	**Nature of water body**	**Geography and area**	**Approximate depth (in m)**
Morni Hills Tikkar Taal Lake, Panchkula	Natural Lake	Himalaya foothill (0.25 km^2)^	10.0
Tilyar Lake, Rohtak	Lake	Plain (Area 0.53 km^2^)	5.0
Jawaharlal Nehru canal, Rohtak	Canal	Plain (Area N.A.)	8.0
Kharawad Village Well, Rohtak	Well	Plain (Area N.A.)	3.0
Suraj Kund, Faridabad	Pond	Aravli foothill (0.22 km^2^)	4.0

**Table 2:** Temperature at the time of sample collection.

**Water body**	**Water temperature at the time of sample collection**			
	**Winter (December 2013) (°C)**	**Spring (March 2014) (°C)**	**Summer (June 2014) (°C)**	**Autumn (September 2014) (°C)**
Morni Hills Tikkar Taal Lake, Panchkula	10	23	38	27
Tilyar Lake, Rohtak	21	25	42	39
Jawaharlal Nehru canal, Rohtak	21	25	42	39
Kharawad Village Well, Rohtak	19	24	43	32
Suraj Kund, Faridabad	24	28	43	41

**Table 3:** The pH values of the water samples in different seasons.

**Water body**	**Winter (December 2013)**	**Spring (March 2014)**	**Summer (June 2014)**	**Autumn (September 2014)**
Morni Hills Tikker Tal Lake, Panchkula	6.35	7.14	7.58	7.67
Tilyar Lake, Rohtak	9.56	8.00	9.74	8.82
Jawaharlal Nehru canal, Rohtak	7.67	7.62	7.77	7.83
Kharawad village well, Rohtak	7.97	8.14	7.78	7.89
Suraj Kund, Faridabad	7.81	8.01	8.26	8.12

**Table 4:** Overall diatoms distribution in five water bodies.

**Diatom**	**Water Body**				
	**Tikkar Taal Lake, Panchkula**	**Tilyar Lake, Rohtak**	**Jawahar Lal Nehru Canal, Rohtak**	**Kharawad Well, Rohtak**	**Surujkund, Faridabad**
*Achnanthes*	**+**	−	−	−	−
*Amphora*	−	−	**+**	−	−
*Aulacoseira*	−	**+**	−	−	−
*Cocconeis*	−	**+**	**+**	−	−
*Cyclotella*	**+**	**+**	**+**	**+**	**+**
*Cymatopleura*	−	−	**+**	−	−
*Cymbella*	−	**+**	**+**	**+**	**+**
*Diploneis*	−	−	**+**	−	−
*Diatoma*	−	**+**	**+**	−	−
*Epithemia*	−	**+**	**+**	−	−
*Eunotia*	−	**+**	**+**	−	−
*Fragillaria*	−	−	−	−	**+**
*Gomphonema*	**+**	−	−	−	**+**
*Hantzschia*	**+**	−	−	−	−
*Gyrosigma*	−	**+**	**+**	−	−
*Melosira*	−	**+**	**+**	−	−
*Navicula*	**+**	**+**	**+**	**+**	**+**
*Nitzschia*	**+**	**+**	**+**	**+**	**+**
*Pinnularia*	−	**+**	**+**	−	−
*Pseudostaurosira*	**+**	−	−	−	−
*Stauroneis*	**+**	−	−	−	−
*Rhoicosphenia*	−	**+**	**+**	−	−
*Staurosirella*	−	−	**+**	−	−
*Surirella*	−	−	−	−	**+**
*Synedra*	−	**+**	**+**	−	−

*Symbol description*: (+ Present; − Absent).
