# Supplementary material for: A systematic databasing of diatoms from different geographical localities and sites of Haryana for advancing validation of forensic diatomology
Source: Data Brief. 2016 Nov 24;10:63–8. doi: 10.1016/j.dib.2016.11.072 (PMC5137176; doi:10.1016/j.dib.2016.11.072)
Supplement: Supplementary file 1 — Supplementary material [file mmc2.zip › Figures.doc]

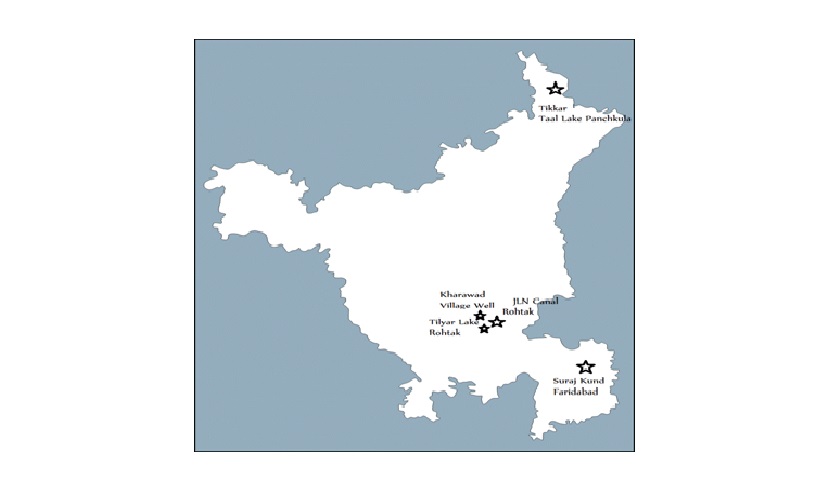


**Fig. 1 Map of Haryana showing location of selected water bodies**

| 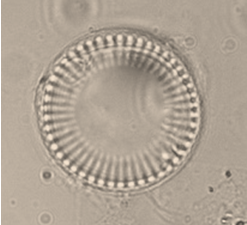 | 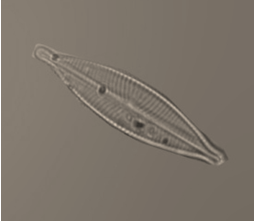 | 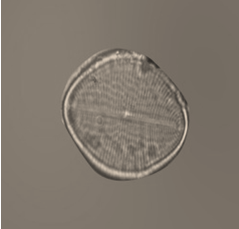 |
| --- | --- | --- |
| ***Cyclotella sp.*** | ***Navicula sp.*** | ***Cocconeis sp.*** |
| 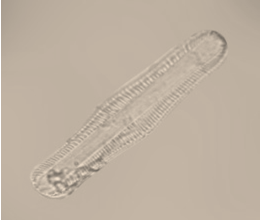 | 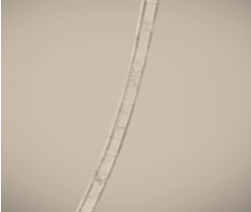 | 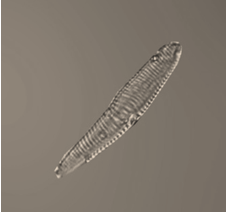 |
| ***Pinnularia sp.*** | ***Melosira sp.*** | ***Eunotia sp.*** |
| 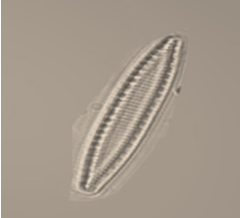 | 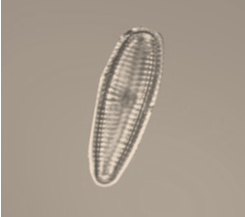 | 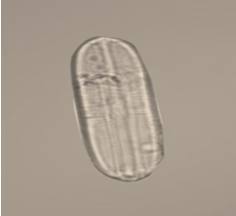 |
| ***Nitzschia sp.*** | ***Gomphonema sp.*** | ***Diploneis sp.*** |
| 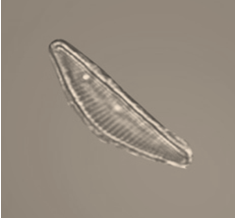 | 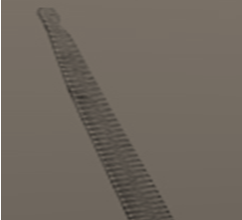 | 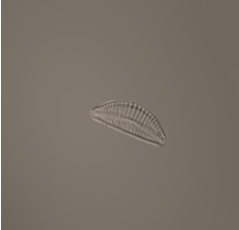 |
| ***Cymbella sp.*** | ***Synedra sp.*** | ***Epithemia sp.*** |

**Fig. 2 Photomicrographs of diatoms grabbed through light microscope**
